# Radiation oncology alternative payment model to medical physics profession: More benefits than detriments

**DOI:** 10.1002/acm2.12782

**Published:** 2019-12-13

**Authors:** Todd Pawlicki, Eric Ford, Yi Rong

**Affiliations:** ^1^ Department of Radiation Medicine and Applied Sciences University of California San Diego La Jolla CA; ^2^ Department of Radiation Oncology University of Washington Medical Center Seattle WA; ^3^ Department of Radiation Oncology University of California‐Davis Cancer Center Sacramento CA

**Abbreviations**
AAPM: American Association of Physicists in Medicine
AI/ML: Artificial Intelligence/Machine Learning
ASTRO: American Society of Radiation Oncology
CMMS: Centers for Medicare & Medicaid Services
CTV: clinical tumor volume
GTV: gross tumor volume
IGRT: Image Guided Radiotherapy
IMRT: Intensity Modulated Radiotherapy
MLCs: Multi‐leaf Collimators
PTV: planning tumor volume
RO‐APM: Radiation Oncology Alternative Payment Model
RO‐ILS: Radiation Oncology Incident Learning System
SGRT: Surface Guided Radiotherapy.

## 1 INTRODUCTION

Centers for Medicare & Medicaid Services (CMMS) proposed a “Radiation Oncology Alternative Payment Model” (RO‐APM) in July 2019, as a major step for transitioning from volume‐based to value‐based healthcare.^1^ In September 2019, ASTRO submitted comments to CMMS addressing their appreciation and concerns with the APM.^2^ The Chair of ASTRO Board of Directors, Dr. Paul Harari, has pointed out three key elements for CMMS to consider in the process of payment reform, including (i) rewarding radiation oncologists for treatment quality improvement, (ii) ensuring fair predictable payment for radiation oncologists, and (iii) incentivizing quality care that is associated with improved patient outcomes.^2^ The proposed payment reforms have created excited discussions among our fellow radiation oncologists, but not so much discussion among medical physicists. Perhaps our lack of participation is because we might not realize the significant impact this billing model reform might bring to our profession. Specifically, will this payment reform that is based on “value" bring more recognition and job opportunities to Medical Physicists? Or will it be detrimental to our profession since the fundamental goal of the reform is to reduce healthcare costs provided that physicists’ services have been insufficiently supported by appropriate billing codes? Herein, we invited two well‐established medical physicists who have done extensive work in promoting the medical physics profession and improving quality/safety for radiation medicine. Dr. Todd Pawlicki is arguing for the proposition that “*RO‐APM brings more benefits than detriments to the medical physics profession*”, while Dr. Eric Ford is arguing against it.

Dr. Todd Pawlicki is Professor and Vice‐Chair of Medical Physics in the Department of Radiation Medicine and Applied Sciences, University of California, San Diego. He has been focused on translating quality improvement techniques from engineering and manufacturing to radiation oncology since 2004 and has several publications, book chapters, and a book on the topic of quality and safety. His current AAPM participation includes serving as Chair of the Management of Medical Physics Programs and Departments Sub‐committee, Vice Chair of the Medical Physics 3.0 Working Group, and as a member of TG‐332, TG‐302, and TG‐288. He is also a founding partner of TreatSafely Foundation; a non‐profit organization created to improve quality and safety in radiation medicine through training, education, and peer‐to‐peer dissemination of best practices.

Dr. Eric Ford is Professor and Interim Director and Vice Chair of Medical Physics at University of Washington, Seattle. He trained in medical physics at Memorial Sloan‐Kettering and was on the faculty of Johns Hopkins for 7 years. There he helped develop novel image‐guided radiation platform for laboratory work and published the first study in radiation oncology using the prospective risk analysis method of failure mode and effects analysis. More recently he has been involved in global oncology efforts to improve access to care. Dr. Ford serves on the board of directors of AAPM, chairs an AAPM Task Group, and chairs the Clinical Affairs and Quality Committee in ASTRO.

## 2 OPENING STATEMENTS

### 2.1 Todd Pawlicki, PhD

The RO‐APM led by the CMMS aims to improve quality and control cost of healthcare by shifting from a fee‐for‐service to an episode‐of‐care reimbursement model. While the final configuration of the RO‐APM is not finalized, it is very clear that the U.S. health care system must move in this direction to control costs or risk a lowering GDP, lowering employment, and increasing inflation.

A change to the RO‐APM is likely to result in a further emphasis on efficiency and cost reductions to maintain radiation oncology profit margins. This focus could lead to a reduction in staffing levels (including medical physicists) and have serious deleterious effects on treatment quality and patient safety. Physicists could easily be replaced with less qualified (and less expensive) staff that would be tasked with the majority of technical/routine work, leaving only a very few qualified physicists in the department for key physics‐only work such as output calibrations. Achieving further operational efficiency might be realized by an increased use of hypo‐fractionated treatments, which are more complex and risky than conventionally fractionated treatments. Paradoxically, when a physicist’s expertise needed most, the medical physics profession might be contracting. In addition to all this, the RO‐APM could stifle innovation in radiation oncology because of the technical expertise will not exist in the department to develop and implement it; not to mention that hospital administration will not have the financial incentive to do so. It is a bleak outlook when you stop to think about it.

How can I argue *for* the proposition? To answer that question, we need to go back a ways. I started working in radiation oncology back in 1992 – before MLCs, IMRT, IGRT, SGRT, AI/ML, and all the rest of the novel or once novel technologies that we have now. Fast forward about 10 years when I started doing clinical research in quality and safety, moved to UC San Diego to build and lead a team of physicists and by necessity, stepped back from routine clinical work. From these perspectives, two ideas crystalized for me: 1) the only way to create a large improvement in quality and safety is to change the system of care – new technologies or process tweaks will not reach that goal, and 2) the level of education, training, and clinical acumen of medical physicists entering the field today is so consistently high, compared to when I started, that the current clinical role of the medical physicist drastically undervalues their potential impact on patient care.

With these two axioms in mind, I can confidently say that the RO‐APM would be *much more* of a benefit to the medical physics profession than it is a detriment – provided that physicists are willing to transform their current clinical role. This transformation will be easier if the physicist’s work is not defined by specific billing codes needed for reimbursement. The RO‐APM would remove this barrier and allow flexibility in the physicist’s job functions.

What does the transformation look like? The new medical physicist role will have primary responsibility for all technical decisions related to patient care in radiation oncology including the management of the patient’s care path through radiation treatment. After the decision has been made by the radiation oncologist to use radiotherapy, the physicist will then lead the radiation treatment team, collaborating with the radiation oncologist, to determine and deliver the best radiation treatment. The radiation oncologist will make the decision of whether or not radiation should be used to treat the patient’s disease, CTV delineation, managing short‐ and long‐term complications, as well as seeing any patient with a suspected cancer diagnosis to coordinate that patient’s care – even for those who do not require radiation therapy. Shared decisions by the radiation oncologist and physicist will be related to secondary imaging and GTV delineation, total dose, and fractionation.

Patient‐specific decisions made by the medical physicist will include: patient immobilization and PTV delineation, patient imaging for setup and inter‐ and intra‐fraction monitoring, type and approach of radiation delivery, the necessary steps to ensure quality and safety of the treatment plan, and treatment delivery. The physicist will liaise with other physicians (e.g., medical, surgical, referring physicians) and their support staff as necessary to facilitate a patient’s care. Technological innovations will be alive and well in this new paradigm because the physicist will determine how and when to implement them. Innovation of new hardware and software technologies will occur via the traditional pathway of securing extramural funding from vendors, private foundations, or the government to support that development; just as the radiation oncologist basic science researchers do.

Controlling cost while maintaining or improving quality and patient safety will require this transformation of the medical physicist’s (and radiation oncologist’s) role. In direct collaboration with the radiation oncologist, the physicist will be a central part of team‐based care in a value‐based care model that is responsible for optimizing key business aspects of a well‐functioning practice (patient census, clinical workflow, and development of quality improvement and patient safety initiatives). Ultimately, the RO‐APM will be a great benefit to the medical physics profession.

### 2.2 Eric Ford, PhD

The potential effects of the RO‐APM are difficult to judge given its complexity and the fact that this will be the first mandatory APM for any medical specialty. One stated goal of the CMMS is to cut costs. Therefore, one might predict that one effect of this new model is lower overall support paid out, at first for Medicare patient but eventually also for private payers (insurance companies). The overall result is a pressure to reduce costs. Of this there is little doubt. This might lead to reduced staffing of medical physicists, increased reliance on technical staff who are not qualified medical physicists, or reduced support for medical physics activities in other ways. Thus, medical physicists have a well‐founded anxiety about the potential impacts of the RO‐APM system.

The effects of the RO‐APM may be compounded by other factors as well. One likely outcome of the RO‐APM is an increased reliance on hypo‐fractionated regimens (as in single‐payer systems like Canada and Europe). Deploying such services safely, however, typically requires more physics support, not less. The RO‐APM also comes at a time where automation is being increasingly used. This may impact medical physicists. The commissioning of a linac, typically, is thought to require 8–12 weeks of intensive physics time, but now at least one vendor is advertising a system with a commissioning time of two days. This unit also includes automated system for quality assurance. Automation may mean that more routine parts of the medical physicist’s job are quickly taken over. There will likely be other cost pressures on the healthcare system as well. Some models have suggested that the RO‐APM will require that *more* support staff be hired to manage the administration of this complex system. These higher costs may drain resources away from medical physics.

Given these pressures, it is difficult to see how the RO‐APM might improve the lot of medical physics profession. Perhaps an enlightened administrator or other decision maker when confronted with the new model might take a new look at established safety recommendations such as the Safety is No Accident report from ASTRO.^3^ This might move them to retain or even increase support to medical physics. Or perhaps this decision maker might consider new roles for medical physicists beyond routine tasks (e.g. a medical physicist hired to be an in‐house expert on automated treatment planning). While this may well happen in a handful of centers (as indeed is already happening), the reality is that for many administrators it will be far simpler to reduce costs by simply cutting physics support.

Consider also the signals that administrators and decision makers are receiving. An example is the separation of reimbursements into professional fees and technical fees (in radiation oncology typically 20% of the reimbursements are billed by clinicians as professional fees while the rest are technical). This split system has existed for many years and will continue to exist under the RO‐APM. It has resulted in a symbiotic interdependency between clinical providers and administrators. Because the revenue from professional fees was never enough to support the salaries of clinicians the two parts became intimately dependent. This sends a signal of sorts. If this signal could be decoded it might read something like this: “[Memo from CMMS to Hospital X]: Do not back off on your support for physicians. Remember we are paying for this support.” No such signal exists for the medical physicist. There is nothing baked into the system that specifically recognizes their value.

In the end we are all a team and these divisions may not matter much (or should not matter). However, this is predicated on the fact that a functional and effective team is in place at each center. Will the new system reward and support such teamwork? In some ways it certainly will. If, for example, your team can achieve a lower rate of re‐hospitalization you will be rewarded. If your team participates in a quality improvement program under a Patient Safety Organization like RO‐ILS: Radiation Oncology Incident Learning System, you will be rewarded. And so on. In other ways, however, it does not. If the business entities were to undertake extreme cost‐cutting measures, the system would have little to say about it until the quality metrics fell below some level. That might be acceptable. After all, quality of care is what we are after. However, quality metrics are notoriously difficult to measure, especially in oncology. The result is that a wide drift in quality may be allowed in the new system and there may not be enough backstops to the cost pressures to prevent this.

There are many things that are unclear about the new RO‐APM system, but the danger for medical physicists is that it makes them into the equivalent of the Greatest American Hero (1981‐1983). In this show, Ralph Hinkley, an LA public school teacher, is visited by aliens while on a school field trip. The aliens give him a superhero suit which allows him to fly and do amazing feats, but they leave him little else in the way of support. (He has an instruction book for the suit, but he loses it). Without this support he is “flying away wing and a prayer.” Is it me?

## 3 REBUTTAL

### 3.1 Todd Pawlicki, PhD

There are a number of points where Dr. Ford and I agree about the future impact of the RO‐APM such as the pressure to reduce costs, the risk of reducing medical physics staffing levels, the likely reliance on hypo‐fractionated regimens (that require more, not less, physics support), and the potential increase in the number of administrators to deal with the complexity of the RO‐APM that will in turn require hospitals to find savings anywhere they can. As Dr. Ford mentions, other factors of downward pressure on physics staffing levels are due to streamlined linac commissioning, more efficient QA, and automation in general. The prospect of losing physics jobs to automation is particularly frustrating since physicists are the ones largely responsible for either developing or implementing those technologies. Furthermore, Dr. Ford correctly points out that the interdependency between physicians and administrators related to the mechanisms of reimbursement from CMMS is a real issue that can lead to undervaluing the role of the medical physicist. In my experience, this already occurs to some degree and the RO‐APM would only exacerbate the issue.

The medical physicist’s response to these professional threats is critically important. The challenge for physicists is to worry less about “protecting our turf” and to put more focus on how physicists can add even more value to patient care than they currently are. From this perspective, the RO‐APM (and automation) provides a unique opportunity to make a revolutionary change in the role of the medical physicist. Physicists should be automatous decision makers about how patients are treated with radiation therapy.

There is a sense of value in being recognized as the departmental expert who ensures treatment quality and patient safety, but this can also be seen as limiting the medical physicist’s role in patient care and therefore a risky professional position to be in. First of all, quality and safety is everyone’s responsibility, not just the physicist. And second, no other department in the hospital has a fulltime role for someone with an advanced degree to focus on quality and safety. It is understandable why upper level hospital administrators might question the expense of medical physicists.

The only point of disagreement that I have with Dr. Ford’s opening statement is the potential for a wide drift in quality (and patient safety) with the RO‐APM. This may be true only if we use the same workflows and job roles as we are currently using. If physicists redefine their role and responsibilities together with some additional training, they can dramatically improve quality and patient safety in radiation oncology while also improving efficiency and access to radiation therapy services. The challenge is determining exactly what the redefined roles and responsibilities should be and how to make the transition. The AAPM Medical Physics 3.0 Working Group is already focused on the future of medical physicists in radiation oncology (among other goals). For all these reasons, it is likely the RO‐APM will ultimately result in more benefits than detriments to the radiation oncology medical physics profession.

### 3.2 Eric Ford, PhD

Dr. Pawlicki presents persuasive arguments that the RO‐APM might benefit medical physics. The vision is a compelling one – the physicist taking on activities such as “liaise with other physicians” including medical oncologists and surgeons, and performing other lead roles; the medical physicist becoming a sort of “uber‐doctor” responsible for the quality of care. As compelling as this vision, it is not what the RO‐APM calls for, at least not explicitly. The RO‐APM does recognize the role of physicians and their special part (enshrined in the model by way of the professional fees). But there is no such recognition of the professional role of the medical physicist in radiation oncology.

Does this matter? I argue that it does. Physicists need support in order to execute the turn‐about in job description that Dr. Pawlicki calls for. They must be, in his words, “willing to transform their current clinical role”. I would add “being willing *and able*” to do this. Being able to make such a shift requires a supportive environment. Certainly, some medical physicists at some centers will have such an environment and will be able to shift their focus toward becoming officers of overall quality. With the proper support, medical physicists are capable of many things. I don’t argue that. What I argue is that the RO‐APM ruling does not support this. It is focused on the economic problems, that is, delivering quality care while cutting costs. Faced with the pressures to reduce costs, it is difficult to see why many hospital administrators would carve out a special niche for medical physicists when it is expensive to do so.

I believe that the profession might get to a place like Dr. Pawlicki describes, that medical physicists in radiation oncology might become the fully realized “shared decision makers” and a “[leaders of] the radiation treatment team” that he describes. If this happens it will not be because of the RO‐APM, however. It will be because of strong efforts by everyone in the field and initiatives like Medical Physics 3.0 and the Medical Physics Leadership Academy and others. The RO‐APM may be a conveniently timed event that causes the profession to re‐examine itself, but such a re‐examination will likely happen anyway due to the influence of evolving technology and automation. But coincidence is not a cause and in my mind the RO‐APM offers little in the way of support to the profession. If we get to the place that Dr. Pawlicki describes, it will not be because of the RO‐APM, it will be in spite of the RO‐APM.

## CONFLICTS OF INTEREST

The authors declare no conflicts of interest.
